# Monolayer Vanadium‐Doped Tungsten Disulfide: A Room‐Temperature Dilute Magnetic Semiconductor

**DOI:** 10.1002/advs.202001174

**Published:** 2020-11-09

**Authors:** Fu Zhang, Boyang Zheng, Amritanand Sebastian, David H. Olson, Mingzu Liu, Kazunori Fujisawa, Yen Thi Hai Pham, Valery Ortiz Jimenez, Vijaysankar Kalappattil, Leixin Miao, Tianyi Zhang, Rahul Pendurthi, Yu Lei, Ana Laura Elías, Yuanxi Wang, Nasim Alem, Patrick E. Hopkins, Saptarshi Das, Vincent H. Crespi, Manh‐Huong Phan, Mauricio Terrones

**Affiliations:** ^1^ Department of Materials Science and Engineering The Pennsylvania State University University Park PA 16802 USA; ^2^ Center for 2‐ Dimensional and Layered Materials The Pennsylvania State University University Park PA 16802 USA; ^3^ Department of Physics The Pennsylvania State University University Park PA 16802 USA; ^4^ Department of Engineering Science and Mechanics The Pennsylvania State University University Park PA 16802 USA; ^5^ Department of Mechanical and Aerospace Engineering University of Virginia Charlottesville VA 22904 USA; ^6^ Research Initiative for Supra‐Materials Shinshu University 4‐17‐1 Wakasato Nagano 380‐8553 Japan; ^7^ Department of Physics University of South Florida Tampa FL 33620 USA; ^8^ Department of Physics Applied Physics and Astronomy Binghamton University Binghamton NY 13902 USA; ^9^ 2D Crystal Consortium The Pennsylvania State University University Park PA 16802 USA; ^10^ Department of Chemistry The Pennsylvania State University University Park PA 16802 USA

**Keywords:** 2D ferromagnets, dilute magnetic semiconductors, room‐temperature ferromagnetism, tungsten disulfide, vanadium doping

## Abstract

Dilute magnetic semiconductors (DMS), achieved through substitutional doping of spin‐polarized transition metals into semiconducting systems, enable experimental modulation of spin dynamics in ways that hold great promise for novel magneto–electric or magneto–optical devices, especially for two‐dimensional (2D) systems such as transition metal dichalcogenides that accentuate interactions and activate valley degrees of freedom. Practical applications of 2D magnetism will likely require room‐temperature operation, air stability, and (for magnetic semiconductors) the ability to achieve optimal doping levels without dopant aggregation. Here, room‐temperature ferromagnetic order obtained in semiconducting vanadium‐doped tungsten disulfide monolayers produced by a reliable single‐step film sulfidation method across an exceptionally wide range of vanadium concentrations, up to 12 at% with minimal dopant aggregation, is described. These monolayers develop p‐type transport as a function of vanadium incorporation and rapidly reach ambipolarity. Ferromagnetism peaks at an intermediate vanadium concentration of ~2 at% and decreases for higher concentrations, which is consistent with quenching due to orbital hybridization at closer vanadium–vanadium spacings, as supported by transmission electron microscopy, magnetometry, and first‐principles calculations. Room‐temperature 2D‐DMS provide a new component to expand the functional scope of van der Waals heterostructures and bring semiconducting magnetic 2D heterostructures into the realm of practical application.

## Graphical abstract

Room‐temperature ferromagnetism is achieved in semiconducting vanadium‐doped tungsten disulfide monolayers. A reproducible and atmospheric pressure film sulfidation growth method yields doping concentration tunability in air‐stable samples. These monolayers develop p‐type transport as a function of vanadium incorporation and rapidly reach ambipolarity. The ferromagnetic behavior in this dilute semiconductor is modeled and understood through first‐principles calculations.

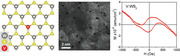

Existing two‐dimensional (2D) ferromagnets are a source of fascinating fundamental physical phenomena^[^ ^1^, ^2^, ^3^, ^4^, ^5^ ^]^ that currently require cryogenic temperatures^[^ ^1^, ^4^ ^]^, protection from the air^[^ ^1^, ^2^, ^3^ ^]^, or a modest external field to induce magnetization^[^ ^4^ ^]^. 2D ferromagnets that are metallic^[^ ^5^ ^]^ cannot access the rich electronic and optical phenomena open to semiconductors. Dilute magnetic semiconductors (DMS) have been realized in bulk III–V systems such as Mn‐doped GaAs, with long efforts toward achieving room‐temperature ferromagnetism in the face of dopant clustering and phase segregation.^[^ ^6^, ^7^, ^8^ ^]^ Pioneering efforts toward achieving DMS behavior in semiconducting transition metal dichalcogenides (TMD)^[^ ^9^, ^10^ ^]^ have recently burgeoned on atomically thin samples toward the introduction of magnetic transition metal ions such as V,^[^ ^11^ ^]^ Ni,^[^ ^12^ ^]^ Co,^[^ ^13^ ^]^ and Mn^[^ ^14^ ^]^ into the host lattice. While first‐principles calculations predict tunable ferromagnetism in V‐doped MoS_2_,^[^ ^15^ ^]^ WS_2_,^[^ ^16^ ^]^ and WSe_2_^[^ ^17^ ^]^ monolayers, the reliable experimental realization of ferromagnetism in thin‐film samples^[^ ^18^ ^]^ has been challenging. Intrinsic ferromagnetism has been confirmed in semiconducting monolayer CrI_3_^[^ ^1^, ^2^, ^3^ ^]^ and insulating few‐layer Cr_2_Ge_2_Te_6_^[^ ^4^ ^]^ at cryogenic temperatures. Moreover, a transition from paramagnetic to ferromagnetic in vanadium diselenide has been recorded when this metallic material was isolated in monolayer form.^[^ ^5^ ^]^ Air sensitive monolayer VSe_2_ has displayed ferromagnetic order even at and above room temperatures.^[^ ^19^ ^]^

Monolayer samples provide compelling advantages in the characterization of atomic structure and integration into van der Waals heterostructures.^[^ ^10^ ^]^ Monolayer tungsten disulfide is a direct‐gap semiconductor with high photoluminescence yield that can achieve a reasonably high on/off current ratio (>10^5^) in field‐effect transistor geometries.^[^ ^20^ ^]^ Reliable substitutional cation doping of WS_2_ and its sister material MoS_2_ can induce degenerate n‐type (rhenium^[^ ^21^ ^]^) and p‐type (carbon^[^ ^22^ ^]^ and niobium^[^ ^23^ ^]^) conduction. Beyond simply introducing charge carriers, a judicious choice of dopant may also introduce spin polarization. Scalable and controllable synthesis of single‐phase monolayer DMSs with ferromagnetic ordering at room temperature could thus provide a new component for van der Waals heterostructures that express novel modes of magneto–electric and magneto–optical response.^[^ ^24^, ^25^ ^]^

We report the single‐step and atmospheric pressure deposition (via film sulfidation) of high‐quality V‐doped WS_2_ monolayers exhibiting room‐temperature ferromagnetism. Aberration‐corrected high‐resolution scanning transmission electron microscopy (AC‐HRSTEM) and X‐ray photoelectron spectroscopy (XPS) reveal substitutional vanadium concentrations up to 12 atomic percent (at%) without substantial structural deformation or degradation. Interestingly, vanadium doping (or alloying) reduces the optical bandgap and induces p‐branch transport that reaches ambipolarity. What appears to be intrinsic ferromagnetic order is achieved at room temperature, with a maximum coercivity and saturation magnetization at an intermediate vanadium concentration (≈2 at%). First‐principles calculations suggest that magnetism can be further strengthened by optimizing the distribution of dopant–dopant neighbor separations and also reveal how spin‐polarized impurity levels break the valley degeneracy. These results now establish a promising route to room‐temperature 2D spintronic devices.

Pristine and V‐doped WS_2_ monolayers were synthesized by chemical vapor deposition (CVD)^[^ ^26^ ^]^ (schematics in **Figure** **1**a) with ammonium metatungstate ((NH_4_)_6_H_2_W_12_O_40_·*x*H_2_O, AMT) and vanadium oxide sulfate (VO[SO_4_]) acting as W/V cation precursors, in conjunction with sodium cholate (C_24_H_39_NaO_5_ ·*x*H_2_O) as a surfactant salt, all dissolved in deionized water. VO[SO_4_] directly supplies V^4+^ ions (i.e., oxovanadium, VO^2+^), which may be the key to reproducibly achieving a wide range of substitutional V concentrations in WS_2_; results for alternative precursors are described in Figure S1, Supporting Information. The prepared solutions were spin‐coated on oxidized Si substrates, followed by sulfidation at 800 °C under atmospheric pressure (Figure 1 and Experimental Section). As‐grown pristine and V‐doped WS_2_ monolayers have regular triangular shapes 10–50 µm across (Figure S2a, Supporting Information). The dopant's elemental fingerprints from transmission electron microscopy's (TEM) electron energy loss spectroscopy (EELS, Figure 1b) at the vanadium L_2,3_ edges of 513 and 521 eV were further confirmed by the observation of a vanadium K*α* peak at ≈4.95 keV in STEM energy‐dispersive X‐ray spectroscopy (STEM/EDS, Figure S2c, Supporting Information), and XPS elemental analyses (Figure S2d–f, Supporting Information). An oxygen peak is unavoidable in the EELS spectra due to oxidation of the sample surface and film support. With the constant overall volume of the precursor solution, the vanadium precursor concentration was varied from zero to 1 × 10^−5^, 1 × 10^−4^, 1 × 10^−3^, and 5 × 10^−3 ^mol L^−1^; (higher vanadium concentrations triggered precipitation upon mixing W and V precursor solutions, leading to degradation of the WS_2_ monolayer's crystallinity, edge regularity, and flatness). XPS analysis measured overall doping levels of ≈1.5 at% and ≈10 at% for the 1 × 10^−4^ and 1 × 10^−3 ^mol L^−1^ solutions, while the vanadium concentration in the 1 × 10^−5^ sample was below the XPS detection limit. Direct enumeration of vanadium dopants by atomic‐resolution TEM (**Figure** **2**a–d; Figure S3a,b, Supporting Information) yields more sensitive results, with average vanadium concentrations of 0.4, 2.0, 8.0, and 12.0 at% obtained for V‐doped WS_2_ grown using the 1 × 10^−5^, 1 × 10^−4^, 1 × 10^−3^, and 5 × 10^−3 ^mol L^−1^ solutions, respectively; no detectable vanadium was found in pristine WS_2_ monolayers grown from 0 mol L^−1^ solution. The 0.4, 2.0, and 8.0 at% samples showed large area monolayer morphology and were subject to further detailed optical, electronic, structural, and magnetic characterization as described below. The TEM images of Figure S4, Supporting Information, show how the vanadium concentration varies from the center to edge of the triangular monolayer flakes, for example, from 5.5 at% at the center to 2.1 and 1.7 at% in the middle and outer regions of a flake grown from 1 × 10^−4^ mol L^−1^ solution; the intermediate (middle) value is used to denote each sample, and this is the region from which photoluminescence (PL) Raman spectroscopy and electrical transport measurements are generally taken.

**Figure 1:**
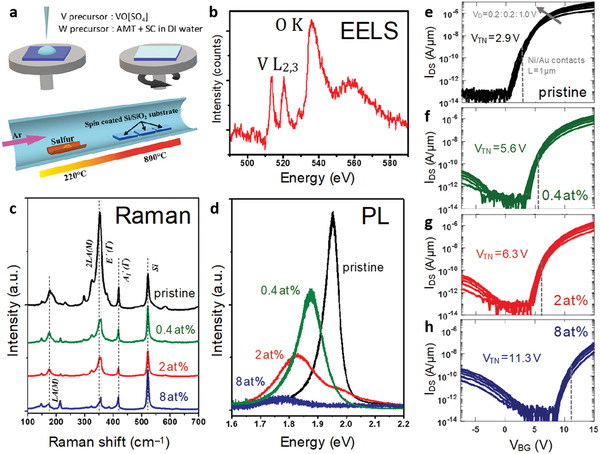
a) One‐step synthesis of monolayer V‐doped WS_2_, optical and electronic properties, as described schematically, b) yields a TEM/EELS spectrum with a prominent vanadium L_2,3_ edge. c) A loss of double resonance in Raman (under 532 nm excitation) and d) pronounced change in photoluminescence response reflect a change of electronic structure as a function of V doping. e–h) Back‐gated V‐doped WS_2_ field‐effect transistors were fabricated on a 50 nm thick Al_2_O_3_ substrate with a Pt/TiN/p^++^ back‐gate electrode for each doping level. Drain current (*I*_DS_) versus back‐gate voltage (*V*_BG_) (obtained for drain voltages from 0.2 to 1 V in 0.2 V steps) show a steady shift in threshold voltage across different doping levels and achieve close‐to‐symmetric ambipolar conduction in heavily doped WS_2_.

**Figure 2:**
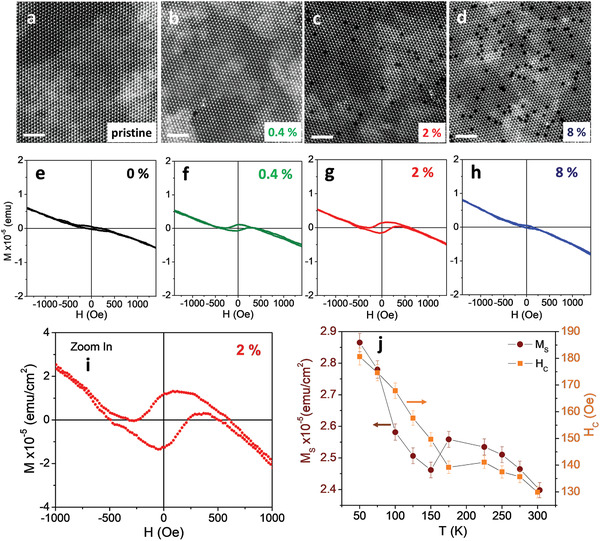
a–d) Atomic resolution HAADF‐STEM images and e–h) magnetization versus field loops at 300 K for pristine and vanadium‐doped WS_2_ monolayers at 0.4, 2, and 8 at% vanadium, scale bars are 2 nm. i) An expanded view of the hysteresis loop for the 2 at% sample and j) its temperature‐dependent saturation magnetization (*M*_S_) and coercivity (*H*_C_).

Representative Raman spectra of pristine and V‐doped WS_2_ monolayers were obtained using excitation wavelengths of 532 nm (Figure 1c) and 488 nm (Figure S5, Supporting Information). Pristine WS_2_ monolayers exhibit the representative E′(Γ) and A_1_′(Γ) first‐order phonon modes at 355 and 417 cm^−1^, respectively.^[^ ^22^ ^]^ Both E′(Γ) and A_1_′(Γ) blueshift as a function of vanadium concentration, which is consistent with previously reported spectra of V‐doped MoS_2_.^[^ ^27^ ^]^ In V‐doped WS_2_ samples, the defect‐activated longitudinal acoustic mode (LA(M)) gradually emerges as the vanadium concentration increases, indicating lattice disorder induced by V dopants.^[^ ^28^ ^]^ A high‐intensity 2LA(M) second‐order double resonance peak involving two longitudinal acoustic phonons^[^ ^29^ ^]^ characteristic for pristine WS_2_ monolayers was progressively suppressed upon increasing vanadium concentration, indicating substantial changes in the WS_2_ electronic structure, which drive the system out of resonance.^[^ ^22^, ^29^ ^]^ Pristine monolayers of WS_2_ show an intense PL peak at 1.97 eV, corresponding to the A exciton^[^ ^30^ ^]^ (Figure 1d). The optical gap decreases under increasing vanadium doping, while the PL peak broadens (likely due to lattice disorder from dopants possibly accompanied by vacancies) and drops in intensity. This evolution of the PL response is consistent with the Raman results discussed above.

Figure 1e–h shows the drain current (*I*_DS_) versus back‐gate voltage (*V*_BG_) transport characteristics for 0, 0.4, 2, and 8 at% vanadium monolayers at various drain voltages (*V*_D_), measured by back‐gated FET geometry (detailed in Experimental Section). The 0 at% device shows unipolar electron conduction with no hole current, as expected for a pristine WS_2_‐based FET. A small hole branch emerges at 0.4 at% doping (Figure 1f), with increasing hole currents at higher doping levels as the threshold voltage shifts from 2.9 volts in the pristine system to 5.6, 6.3, and 11.3 volts in devices with progressively higher doping levels (we extract *V*_TN_ by the iso‐current method for a current of 1 nA µm^−1^ at *V*_D_ = 1 volt). The systematic threshold shift confirms p‐type doping, with the most heavily doped sample demonstrating close‐to‐symmetric ambipolar transport. Strong hole current is not seen as p‐type conduction in most 2D materials, including WS_2_ is limited by a large Schottky barrier (SB) at the metal/WS_2_ interface for hole injection, due to the phenomenon of metal Fermi level pinning. Thermal boundary conductance (*h*_K_) measured at the Al/V‐WS_2_/SiO_2_ interface using time‐domain thermo‐reflectance (Experimental Section, Figure S6, Supporting Information) reveals a significant improvement in heat dissipation under vanadium doping that could be helpful in device applications.

High‐angle annular dark‐field (HAADF)‐STEM imaging confirms the presence of substitutional V atoms at W sites (written V_W_) in WS_2_ and reveals surprisingly little dopant aggregation. Under Z‐contrast imaging, vanadium is easily distinguished from much heavier tungsten, and its concentration can be extracted by statistical analysis of HAADF‐STEM images (Figure 2a–d and Figure S7, Supporting Information) at multiple locations on each flake. Dopant aggregation is modest even at 12 at% vanadium. Occasional aggregations into stripes at high doping levels (Figure S3, Supporting Information) tend to align to the proximate outer edge of the flake, thus suggesting that edge energetics/kinetics influence dopant aggregation.^[^ ^31^ ^]^ Comparing experimental STEM images to image simulations (simulation details in Experimental Section, Figure 2a–d and Figure S7, Supporting Information) clearly reveals elemental identities by contrast differences (W > 2S > V > 1S). At vanadium concentrations of 8 at% or above (Figure S3e, Supporting Information), sulfur vacancies are more likely to be coupled to V atoms (written V_W_+S_vac_), which is consistent with prior work on TMD alloys^[^ ^18^, ^32^ ^]^ and first‐principles calculations described below.

The magnetic properties of pristine and V‐doped WS_2_ monolayer samples were measured by a vibrating sample magnetometer. To exclude unwanted effects on the magnetization versus magnetic field (*M*–*H*) loops that can arise from the SiO_2_ substrate and the double‐sided carbon tape used to hold the sample (see Figure S8, Supporting Information) and from subtracting diamagnetic backgrounds (see Figure S9, Supporting Information),^[^ ^33^, ^34^ ^]^ Figure 2e–h presents smoothed as‐measured *M*–*H* loops at 300 K, and we deduced the saturation magnetization (*M*_S_) and coercive field (*H*_C_) directly from these loops. The pristine WS_2_ sample exhibits a weak ferromagnetic signal, which we tentatively ascribe to undercoordinated sulfur atoms at crystallographic defects (e.g., edges),^[^ ^35^ ^]^ on a diamagnetic background. Vanadium doping of 0.4 at% significantly increases the ferromagnetic signal (*M*_S_ and *H*_C_) with further strengthening at 2 at% doping and then a much weaker ferromagnetic response at 8 at% vanadium. The ferromagnetism observed in V‐doped samples is too strong to originate from edge effects, and its dependence on vanadium concentration suggests an origin in local magnetic moments associated with unpaired electrons in vanadium d orbitals.^[^ ^16^, ^18^, ^33^ ^]^ The 2 at% WS_2_ sample shows large, clear hysteresis loops at all temperatures from which *M*_S_ and *H*_C_ (Figure 2i) are extracted and plotted as a function of temperature (the *M*–*H* loops are close to square when rotated to account for the diamagnetic background). We performed magnetic measurements on three V:WS_2_ samples for each concentration and obtained reproducible results (Figure S10, Supporting Information). The saturation magnetization and coercivity both increase with decreasing temperature (shown in Figure 2j), with an interesting non‐monotonicity of both around 150–200 K. Since magnetic measurements using the Physical Property Measurement System (PPMS) are limited to 350 K, we cannot measure *M*–*T* or *M*–*H* curves at higher temperatures to determine Curie temperature (*T*_C_) values of the V‐WS_2_ samples. In order to estimate the Curie temperature of the V:WS_2_ monolayers (e.g., 2 at% V), we have fitted the *M*(*T*) and *M*_S_(*T*) data to a theoretical expression *M*(*T*) = *M*(0) × [1‐(*T*/*T*_C_)*^*α*^*] adapted for a ferromagnetic system (see Figure S11, Supporting Information).^[^ ^36^, ^37^ ^]^ The value of *T*_C_ ≈ 470 K fitted from the data sets is in line with the previous observation of ferromagnetic signals above 420 K from magnetic force microscopy (MFM) measurements carried out on V‐doped WSe_2_ monolayers.^[^ ^11^ ^]^ Our direct observation of tunable room‐temperature ferromagnetism in the V‐doped WS_2_ monolayers from the magnetometry measurements is an important evidence towards establishing the existence of the ferromagnetic order in V‐doped TMDs.

Estimates of the magnetization per formula unit arising from V doping must address uncertainties in the overall sample thickness, the presence of a diamagnetic background, and possible contributions from point and edge defects as seen in pristine material.^[^ ^35^, ^38^ ^]^ For the most reliable such estimate, we selected the sample with the strongest ferromagnetic signal, at 2 at% V doping, and subtracted from its measured magnetization (≈0.252 × 10^−5^ emu) the highest measured *M*_S_ of pristine‐but‐defective WS_2_ (≈0.241 × 10^−5^ emu). We assumed an average sample thickness of ≈1 nm (i.e., mostly monolayer) across the 3 × 4 mm sample area and estimated the number of formula units from the known mass density (7.5 g cm^−3^) and formula unit mass (245.3 g mol^−1^) of WS_2_ (vanadium does not significantly affect the mass density at this level of precision). The result is an *M*_S_ of ≈0.054 μ_B_ per formula unit or ≈2.7 μ_B_ per V atom; a similar analysis for other samples yields values ranging from 1.5 to 3.6 μ_B_ per V. The variation between samples and modest excess over the computational results (see below) may arise from uncertainties in film thickness (e.g., some multilayer portions on the single‐layer film, possibly with interstitial V^[^ ^39^ ^]^), vanadium concentration (we use the concentration in the middle region of Figure S4, Supporting Information; the exact overall doping level is unknown) and those induced by background subtraction, modulated by the possibly distinct temperature dependences for different magnetic contributions. Considering the role of defects in these systems, it is important to consider evidence that may help distinguish direct contributions arising from the spin polarization of states associated with substitutional vanadium from indirect contributions which might arise from, for example, structural defects that could be induced by the presence of vanadium during growth. The non‐monotonicity in the magnetic response as a function of vanadium doping is helpful here: the return of the magnetic response at the highest vanadium doping levels to behavior similar to that of undoped samples is consistent with the quenching of magnetism observed in first‐principles calculations (see below), but is more difficult to reconcile with an indirect mechanism mediated by structural defects.

Density functional theory calculations show local moments on substitutional vanadium atoms whose spin polarization and coupling are sensitive to the relative placement of dopants, a behavior similar to that seen in other computational investigations of doped TMDs.^[^ ^15^, ^16^, ^40^ ^]^ A single vanadium dopant in a 7 × 7 supercell hosts a local moment of 0.67 μ_B_ with vanadium $d_{z^{2}}$ character associated with a partially occupied spin‐split defect level sitting ≈74 meV below the valence band maximum, as shown in **Figure** **3**. **Table** **1** compiles the results of the interaction between two vanadium dopants in this supercell. At nearest and next‐nearest neighbor separations, the vanadium defect states hybridize strongly, and local moments quench. At larger separations, a clear preference for a ferromagnetic alignment emerges. Estimating the Curie temperature *T*_C_ is beyond the scope of the current paper due to the sensitivity of the coupling parameter *J* to the carrier doping level, quantitative limits of the DFT+U method, and the presence of sulfur vacancies (see Section S13, Supporting Information). In some cases, extracting an effective *J* is not straightforward as one of the spin states is not a local minimum. A very large supercell is also necessary to cover longer range interactions and describe the geometrical frustration of antiferromagnetic interactions. Many‐body interactions such as vanadium triples need to be considered carefully as well. These concerns require a separate computation/theory paper. In this paper, instead of finding a precise *T*_C_, we provide a semiquantitative discussion about the energy scale of interactions. Considering that ferromagnetic order in the experimental system will benefit from additional neighbor interactions beyond the single pair partner in the current model, the ≈10 meV stabilization for parallel alignment over anti‐parallel is reasonably consistent with the observed room‐temperature ferromagnetic order. The origin of the non‐monotonicity in *M*_S_ and *H*_C_ around 150–200 K, where the saturation magnetization drops just as the coercivity more rapidly increases, is less clear. Speculatively, it might reflect domains of competing anti‐ferromagnetic order that reduce *M*_S_ while increasing *H*_C_ through exchange pinning. In spite of all these caveats on the correspondence of computation to experiment, the appearance of similar quenching phenomena even in calculations without spin‐orbit coupling (Table S1, Figure S13, Supporting Information) lends substantial confidence to the quenching mechanism.

**Figure 3:**
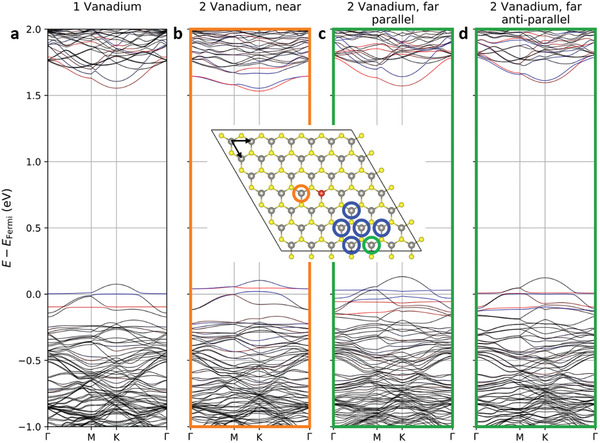
DFT calculation results for V‐doped WS_2_ monolayers. All non‐equivalent positions for the second V dopant are circled. The band structures of a) single vanadium and b–d) two vanadium atoms with the nearest and the farthest separations are plotted. Other, symmetry non‐equivalent *k*‐directions (due to low‐symmetry dopants placements) look similar (Figure S12, Supporting Information). Red/blue indicates spin up/down polarization for states of V $d_{z^{2}}$ character. The arrows in the supercell show the primitive‐cell lattice vectors used to label dopant pairs in Table 1.

**Table 1:** Net moments and energies for vanadium dopant pairs. The energies are relative to that of the pair at the nearest distance. Dopant pairs are labeled by their separation in lattice coordinates and colored in reference to the supercell in Figure 3. Systems were initialized with either parallel or anti‐parallel local moments around the two dopants. Moments after self‐consistent iterations are perpendicular to the plane except for the (0, 2) separation, which is 76° away from this axis. For the closest and next‐closest dopant separations (★), the lowest energy state examined has no spatially resolvable spin texture. “—” means that both parallel and anti‐parallel initial spin textures converge to the same self‐consistent state

Dopant pair in lattice coordinates	Pair separation [Å]	Energy of the most stable spin texture [meV]	Net magnetic moment [μ_B_]	Energy of competing spin texture [meV]	Moment of competing spin texture [μ_B_]
−1, 0	3.19	0 (★)	0.00	—	—
1, 1	5.52	63.9 (★)	0.00	67.5 (⇈)	0.14
0, 2	6.38	48.2 (⇈)	0.93	—	—
1, 2	8.44	71.1 (⇈)	1.18	84.4 (⇅)	0.00
0, 3	9.57	86.8 (⇈)	1.22	95.4 (⇅)	0.03
2, 2	11.02	88.8 (⇈)	1.24	93.6 (⇅)	0.07
1, 3	11.49	86.5 (⇈)	1.23	93.9 (⇅)	0.11

Spin‐momentum locking at the valence band top of WS_2_ interacts closely with the local moments of the vanadium dopants, yielding band crossings or avoided crossings respectively if the spin of a dopant band and the valence band extremum has the same or opposite direction, with the circumstance switching between these two cases as one proceeds from K to −K, which is visible in Figure S12, Supporting Information. For single vanadium (Figure 3a), the two types of spin‐polarized band edges shift in opposite directions in energy such that the valance band maximum (VBM) has an energy difference of 12.6 meV between valleys while the original split in conduction band minima (CBM) collapses in one valley become nearly spin‐degenerate. Increasing the doping level to two vanadium atoms in the supercell (with ferromagnetic order), the VBM difference increases to 19.5 meV, and the CBM shows a uniform spin direction. These band edge offsets arising from the ferromagnetic polarization correspond to an effective external magnetic field on the order of ≈100 T.^[^ ^41^ ^]^

A statistical analysis of the expected fraction of magnetically polarized dopants in a random alloy (Section S11, Supporting Information) suggests that the optimal doping level to obtain maximum saturation magnetization is intermediate between the 2 and 8 at% samples examined experimentally. First‐principles calculations indicate that the nearest‐neighbor separation of a pair of vanadium dopants is 50–90 meV more stable than larger separations, meaning a modest energetic preference for dopant aggregation as is evidenced experimentally by occasional stripes at higher vanadium concentrations. This suggests that the choice of the precursor may be an important factor in atomic‐scale dopant structure optimization (Figure S1, Supporting Information). The rare occurrence of V aggregation in moderately doped samples suggests that synthesis is far from equilibrium, presumably a key to incorporating V dopants into the WS_2_ host in the first place. Finally, the observed spatial correlation between sulfur vacancies and vanadium dopants (Figure S3, Supporting Information) is consistent with computational results that sulfur vacancies bind to single vanadium by ≈0.04 eV and a nearest‐neighbor V–V dimer by ≈0.71 eV.

This study successfully develops a universal, scalable, and controllable synthesis route for V‐doped WS_2_ atomic layers as a dilute magnetic semiconductor, with intrinsic ferromagnetic ordering at room temperature. As the vanadium concentration increases, V‐doped WS_2_ monolayers exhibit a reduction of the optical bandgap and the emergence of p‐type transport, reaching ambipolarity. The vanadium doping induces inherent ferromagnetic ordering at room temperature, with the strongest ferromagnetic signal for the moderately doped (2 at%.) sample among grown samples. The non‐monotonicity of the magnetization as a function of doping level is explained by a combination of atomic resolution TEM imaging and DFT calculations, which show how hybridization between dopant defect states quenches the magnetic moment. An effective Zeeman shift corresponding to ≈100 T is observed in the calculated band structure. Such dilute magnetic semiconductors based on magnetic‐element‐doped transition metal dichalcogenides exhibit great promise as future spintronic/valleytronic devices, with novel magneto–electric and magneto–optical responses. Furthermore, they constitute a new set of atomically thin layers that could be integrated into multifunctional van der Waals heterostructures.

## Experimental Section

##### Synthesis of Pristine and Vanadium‐Doped WS_2_ Monolayers

0.05 g ammonium metatungstate ((NH_4_)_6_H_2_W_12_O_40_·*x*H_2_O, AMT) and 0.2 g sodium cholate (C_24_H_39_NaO_5_·*x*H_2_O) powders were dissolved in 10 mL water to form a tungsten precursor solution. 0.05 g vanadyl sulfate (VO[SO_4_]) powder was dissolved in 10 mL deionized water to form vanadium precursor (1 × 10^−2^ mol L^−1^). The W and V solutions with different concentrations were controlled to form solution‐based cation precursors. The precursor solution was drop‐casted onto an SiO_2_/Si substrate, followed by spin‐coating for 1 min with 3000 rpm. The film sulfidation process was carried out at atmospheric pressure in a quartz reaction tube (1″ inner diameter) with sulfur powder (400 mg) heated upstream at low temperature (220 °C, heated up using a heating tape), and the cation precursor spin‐coated on the SiO_2_/Si substrates, at the high temperature (825 °C) zone. Ultrahigh purity argon was employed as the carrier gas. The furnace was then allowed to cool to room temperature naturally after 15 min synthesis.

##### Materials Characterization

A Renishaw InVia microscope with a Coherent Innova 70C argon‐krypton laser at the excitation of 488 nm and a LabRAM HR evolution (Horiba) equipped with a 532 nm laser were used for acquiring the Raman and photoluminescence (PL) spectra using a backscattering configuration and an 1800^−^ line/mm grating. X‐ray diffraction (XRD) was taken with a PANalytical Empryrean X‐ray diffractometer with a Cu source. X‐ray photoelectron spectroscopy (XPS) experiments were performed using a Physical Electronics VersaProbe II instrument. The binding energy axis was calibrated using sputter cleaned Cu foil (Cu 2p_3/2_ = 932.7 eV, Cu 2p_3/2_ = 75.1 eV). UV–vis absorption spectra were transformed from reflectance measurements, which were acquired on PerkinElmer Lambda 950 with a universal reflectance accessory (URA). STEM‐EDS of the samples was performed in an FEI Talos F200X microscope with a SuperX EDS detector, operating at 200 kV. Aberration‐corrected STEM imaging and EEL spectroscopy were performed using an FEI Titan G^2^ 60–300 microscope, operated at 80 kV with double spherical aberration correction, offering the sub‐angstrom imaging resolution. An HAADF detector with a collection angle of 42–244 mrad, camera length of 115 mm, beam current of 45 pA, and beam convergence of 30 mrad were used for STEM image acquisition. For the HAADF‐STEM images, a Gaussian blur filter (*r* = 2.00) was applied (by the ImageJ program) to eliminate noise and enhance the visibility of structural details, while the line profiles of ADF intensity were captured by analyzing raw STEM images. Atomic resolution STEM image simulations were conducted by using the QSTEM package,^[^ ^42^ ^]^ the case of vanadium dopant coupled with sulfur monovacancy was set to be three symmetric neighboring sulfur monovacancies in the crystal structure to simplify the simulation process. The applied parameters, acceleration voltage, convergence angle, inner/outer angle for the HAADF detector, and spherical aberration (C_3_ and C_5_), were all adjusted according to the experimental conditions.

##### DFT Calculations

Spin‐orbit‐coupled DFT calculations were implemented in the Vienna Ab‐initio Simulation Package (VASP).^[^ ^43^, ^44^, ^45^ ^]^ A 7 × 7 supercell of WS_2_ was tested with different V doping levels. The *z*‐axis cell dimension was 15 Å to isolate a layer from its periodic images. The exchange‐correlation was treated under GGA PBE approximation^[^ ^46^ ^]^ with PAW method.^[^ ^47^ ^]^ The energy cutoff in all calculations was 700 eV, and the *k*‐point sampling was set as 4 × 4 × 1 centered at Γ. The WS_2_ unit cell lattice constant calculated as 3.188 Å matched with previous work^[^ ^48^ ^]^ and was fixed for doped WS_2_ since the doping level was not high enough to change the lattice constant significantly. The residual force after relaxation was smaller than 0.01 eV Å^−1^ for all atoms. All visualizations were done with VESTA^[^ ^49^ ^]^ and pymatgen.^[^ ^50^ ^]^

As the experimental distribution of vanadium dopants was irregular and covered a wide range of pairwise separations, the system was not modeled with a regular array of dopants at uniform mutual separations but instead examined a dopant pair hosted within a large 7 × 7 supercell across a range of separations so that their interactions could be elucidated on a pairwise basis. The two dopants within this supercell were closer to each other than to any periodic replicas. Both ferromagnetic (parallel) and antiferromagnetic (anti‐parallel) initial spin configurations were considered for two vanadium dopants in a supercell, with the system also being able to converge self‐consistently into an unpolarized state in the case of moment quenching.

##### Electronic Device Fabrication

Pristine and V‐doped WS_2_ triangles were transferred from the growth substrate (Si/SiO_2_) to a 50 nm thick and atomic layer deposition grown Al_2_O_3_ substrate with Pt/TiN/p^++^Si as the back‐gate electrode. All FETs were fabricated with a channel length of 1 µm with 40 nm Ni/30 nm Au as the source/drain contact electrodes defined using a standard electron‐beam lithography process.

##### Thermal Transport Measurements

To examine the thermal boundary conductances (*h*_K_) of devices contingent on the use of doped WS_2_, a nominally 80 nm Al film was deposited via electron beam evaporation. The total conductance of the Al/doped WS_2_/SiO_2_ interface was measured via time‐domain thermoreflectance. The specific analyses can be found elsewhere.^[^ ^51^ ^]^ In the implementation, the 808.5 nm output of a Ti:Sapphire oscillator was spectrally separated into high‐energy pump and low‐energy probe paths. The pump was electro‐optically modulated at 8.4 MHz and created a frequency‐dependent heating event at the sample surface. The probe was mechanically delayed in time, and the change in reflectivity due to the pump‐induced heating event (i.e., thermoreflectivity) was monitored as a function of delay time. Both the pump and probe were concentrically focused through a 10× objective, yielding 1/e^2^ diameters of 14 and 11 µm, respectively. The data were fit to the radially symmetric heat diffusion equation to extract the conductances at the Al/doped WS_2_/SiO_2_ interface.

##### Magnetic Measurements

Temperature‐ and magnetic field‐dependent magnetization measurements were carried out in a Physical Property Measurement System (PPMS) from Quantum Design with a vibrating sample magnetometer (VSM) from 2 to 350 K in fields up to 9 T. All magnetic measurements were conducted with the applied magnetic field parallel to the film plane. For temperature‐dependent magnetization measurements (*M*–*T*), the sample was cooled from 350 to 10 K in the presence of a dc magnetic field (0.05 T), and *M*–*T* data were collected from 10 to 350 K following a field‐cooling (FC) protocol. For field‐dependent magnetization measurements (*M*–*H*), the sample was cooled from 300 K in the absence of a dc field, and *M*–*H* data was taken at each selected measurement temperature. The *M*–*H* dependences of an SiO_2_ substrate and a double‐sided carbon tape used to hold the pristine WS_2_ and V‐WSe_2_ samples were also examined to characterize the magnetic background (see Figure S8, Supporting Information). To best reflect the magnetic characteristics of the V‐WS_2_ samples, the as‐measured *M*–*H* loops were showed at 300 K (Figure 2i), and *M*_S_ and *H*_C_ values were deduced directly from these loops after smoothing (Figure S9, Supporting Information). The *M*_S_ values (μ_B_ per formula unit) of the V‐doped WS_2_ monolayers were estimated by carefully subtracting the defect‐induced magnetization contribution from the pristine WS_2_ monolayer.

## Conflict of Interest

The authors declare no conflict of interest.

## Author Contributions

F.Z., T.Z., and M.L. conducted the synthesis experiments. F.Z. collected the TEM data; and F.Z., K.F., L.M., and N.A. performed TEM data analyses. Optical characterization was performed by F.Z. and T.Z.; B.Z., Y.W., and V.C. carried out with the computational calculations, Y.T.H.P., V.K., V.O.J., and M.H.P. performed magnetic measurements and analyzed the magnetic data. A.S., R.P., and S.D. integrated the devices and conducted electronic transport measurement. D.H.O. and P.E.H. carried out with thermal transport measurement. K.F., Y.L., and A.L.E. helped with data analysis, discussion, and guidance about doping precursors and experimental parameters. M.T., M.H.P., and V.C. supervised the work. The manuscript was written through contributions from all and all authors have given approval to the final version of the manuscript.

## Supporting information

Supporting Information
