# Supplementary material for: Monolayer Vanadium‐Doped Tungsten Disulfide: A Room‐Temperature Dilute Magnetic Semiconductor
Source: Adv Sci (Weinh). 2020 Nov 9;7(24):2001174. doi: 10.1002/advs.202001174 (PMC7740087; doi:10.1002/advs.202001174)
Supplement: Supplementary file 1 — Supporting Information [file ADVS-7-2001174-s001.pdf]

© 2020 Wiley-VCH GmbH

ADVANCED SCIENCE
Open Access

Supporting Information

for *Adv. Sci.,* DOI: 10.1002/advs.202001174

Monolayer Vanadium-Doped Tungsten Disulfide: A Room-Temperature Dilute Magnetic Semiconductor

Fu Zhang, Boyang Zheng, Amritanand Sebastian, David H. Olson, Mingzu Liu, Kazunori Fujisawa, Yen Thi Hai Pham, Valery Ortiz Jimenez, Vijaysankar Kalappattil, Leixin Miao, Tianyi Zhang, Rahul Pendurthi, Yu Lei, Ana Laura Elías, Yuanxi Wang, Nasim Alem, Patrick E. Hopkins, Saptarshi Das, Vincent H. Crespi, Manh-Huong Phan,* and Mauricio Terrones**

Monolayer Vanadium-doped Tungsten Disulfide: A Room-Temperature Dilute Magnetic Semiconductor

Fu Zhang[1,2], Boyang Zheng[3], Amritanand Sebastian[4], Hans Olson[5], Mingzu Liu[2,3], Kazunori Fujisawa[2,3,6], Yen Thi Hai Pham[7], Valery Ortiz Jimenez[7], Vijaysankar Kalappattil[7], Leixin Miao[1], Tianyi Zhang[1], Rahul Pendurthi[4], Yu Lei[1,2], Ana Laura Elías[2,3,8], Yuanxi Wang[2,3,9], Nasim Alem[1], Patrick E. Hopkins[5], Saptarshi Das[1,4], Vincent H. Crespi[3,9*], Manh-Huong Phan[7*], Mauricio Terrones[1,2,3,10*]

[1]Department of Materials Science and Engineering, The Pennsylvania State University, University Park, PA 16802, USA

[2]Center for 2- Dimensional and Layered Materials, The Pennsylvania State University, University Park, PA 16802, USA

[3]Department of Physics, The Pennsylvania State University, University Park, PA 16802 USA

[4]Engineering Science and Mechanics, The Pennsylvania State University, University Park, PA 16802, USA

[5]Department of Mechanical and Aerospace Engineering, University of Virginia, Charlottesville, VA 22904, USA

[6]Research Initiative for Supra-Materials, Shinshu University, 4-17-1 Wakasato, Nagano 380-8553, Japan

[7]Department of Physics, University of South Florida, Tampa, Florida 33620, USA

[8]Department of Physics, Binghamton University, Binghamton, NY 13902, USA

[9]2D Crystal Consortium, The Pennsylvania State University, University Park, PA 16802, USA

[10]Department of Chemistry, The Pennsylvania State University, University Park, PA 16802, USA

***Corresponding authors:** mut11@psu.edu, vhc2@psu.edu, phanm@usf.edu*

This file includes:

Supplementary Figures S1-13

Supplementary Tables S1-2

Supplementary Materials

1. Discussion of several V doping precursors for V-doped WS_2 monolayers synthesis.

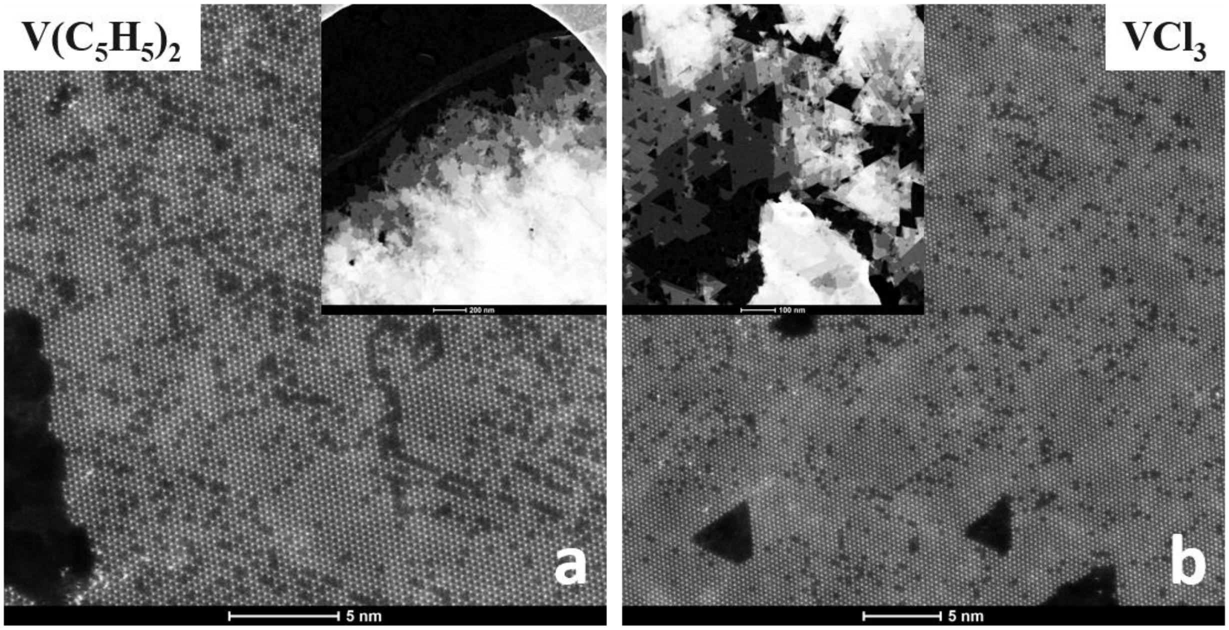

Figure S1 HAADF-STEM image of V-doped WS_2 by different V precursors: (a) $V(C_5H_5)_2$ and (b) VCl_3.

We have studied several vanadium precursors for doping atoms into WS_2 monolayers,

1. For vanadocene (II) ($V(C_5H_5)_2$), we realize doping by powder vaporization growth;[1] it could be doped into the lattice with high concentration; however, there are carbon contaminations or even a defective graphene layers underlying as-grown TMDs, as reported for other metal-organic precursors.[2] The morphology degrades, meaning it fails to form large-area monolayer triangles. Atomic-resolution HAADF-STEM images of highly doped materials show extensive stripes of V dopants in the lattice (**Figure S1**(a)), which substantially affects the physical properties.

2. For vanadium (III) chloride (VCl_3), we realize doping by powder vaporization growth;[1] as this precursor is very air-sensitive, it is handled inside a glove box. Doping could also be realized at high concentration, but the final morphology shows as few-layer WS_2 filled with etched holes probably related to the chloride (Figure S1(b)).

3. Vanadium (IV) oxide sulfate ($VO[SO_4]$) is our primary current focus; with different concentrations of the precursor, we can dope into TMDs as high as 12% of V with good morphology control.

4. Vanadium (V) oxide (V_2O_5) does not work due to high stability and extremely low vapor pressure impeding sulfurization, at least for sulfur vaporization.

5. Ammonium metavanadate (V) (NH_4VO_3) can be dissolved in DI water for spin coating; we have not tried this precursor, but it has been reported by a recent study,[3] which claimed high-concentration doping is possible, yet high-quality large-area monolayer triangles were not found for higher doping concentration.

The distribution of the transition metal dopants in the host TMD lattice is also highly dependent on the synthesis process, as the kinetically driven CVD process may result in segregation and stripe formation of V dopants. Vanadium segregation and striping were consistently detected when using vanadocene ($V(C_5H_5)_2$) and vanadium (III) chloride (VCl_3) as precursors, which would significantly affect the magnetic properties of the materials as revealed by DFT calculations.

We used a nitrogen box to transfer samples between characterization facilities and kept them in a vacuum/glove box for long-term storage. Although samples with higher vanadium concentration maybe more air-sensitive than undoped samples, optical microscopy, photoluminescence, and VSM measurements repeated one week after the first measurements showed negligible variations. The $VO[SO_4]$ precursor is not air sensitive.

2. Elemental analysis and binding energy information V-doped WS_2 monolayers synthesis.

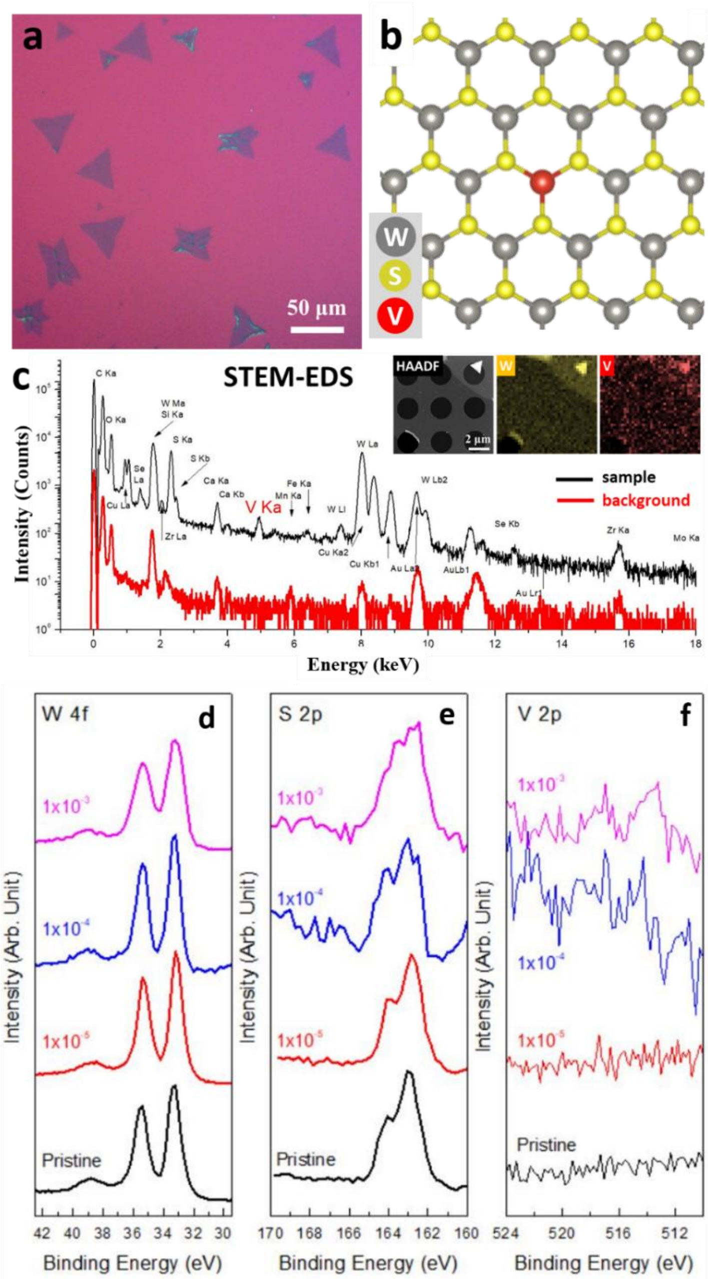

Figure S2 Material characterization of V-doped WS_2. (a) Optical microscope image of triangular V-doped WS_2 monolayers; (b) Structure schematic of one V atom substitutional doping in WS_2 hexagonal lattice; (c) STEM/EDS spectrum of the monolayer V-doped WS_2, inset showing the STEM/EDS elemental mappings of the materials. X-ray photoelectron spectroscopy (XPS) elemental analyses of pristine WS_2 and V-doped WS_2, (d) W 4f, (e) C 1s, and (f) V 2p core levels. The vanadium doping levels were below the XPS detection limit for 1×10^{-5} mol/L (V precursor concentration) sample, and approximately 1.5 at% and 10 at% for the 1×10^{-4} and 1×10^{-3} mol/L samples.

3. Vanadium configuration in the WS_2 monolayer lattice.

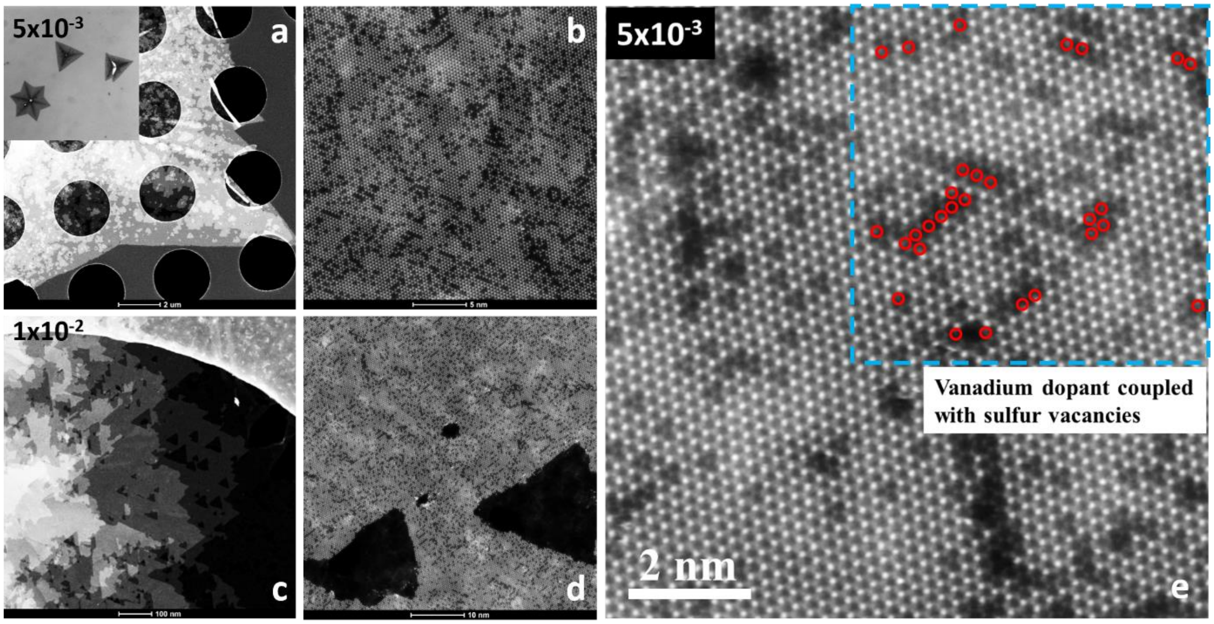

Figure S3 HAADF-STEM images of higher V-doped WS_2 monolayers at V precursor concentration of (a) and (b) 5×10^{-3} mol/L (doping level around 12 %) and (c) and (d) 1×10^{-2} mol/L, respectively; (e) indexing of sulfur monovacancies by red circles, it is clear that the sulfur monovacancies tends to couple with V substitute dopants and even double V substitute dopants.

A vanadium dopant concentration gradient was observed by TEM from this single-step synthesis route, local TEM images in **Figure S4** show concentration gradients from the edge, middle to the center of the triangles, for V-WS_2 grown using the 1×10^{-5}, 1×10^{-4}, and 1×10^{-3} mol/L solutions. As analyzed from TEM images, the concentration (by 1×10^{-4} mol/L solution, medium doped) from the center, middle to the outside area is 5.5 at%, 2.1 at% and 1.7 at%, respectively. The average vanadium concentration used in the main text refers to the middle area local vanadium doping concentration. The corresponding PL and Raman spectroscopy and electrical transport measurements are also conducted in the middle area of the triangles.

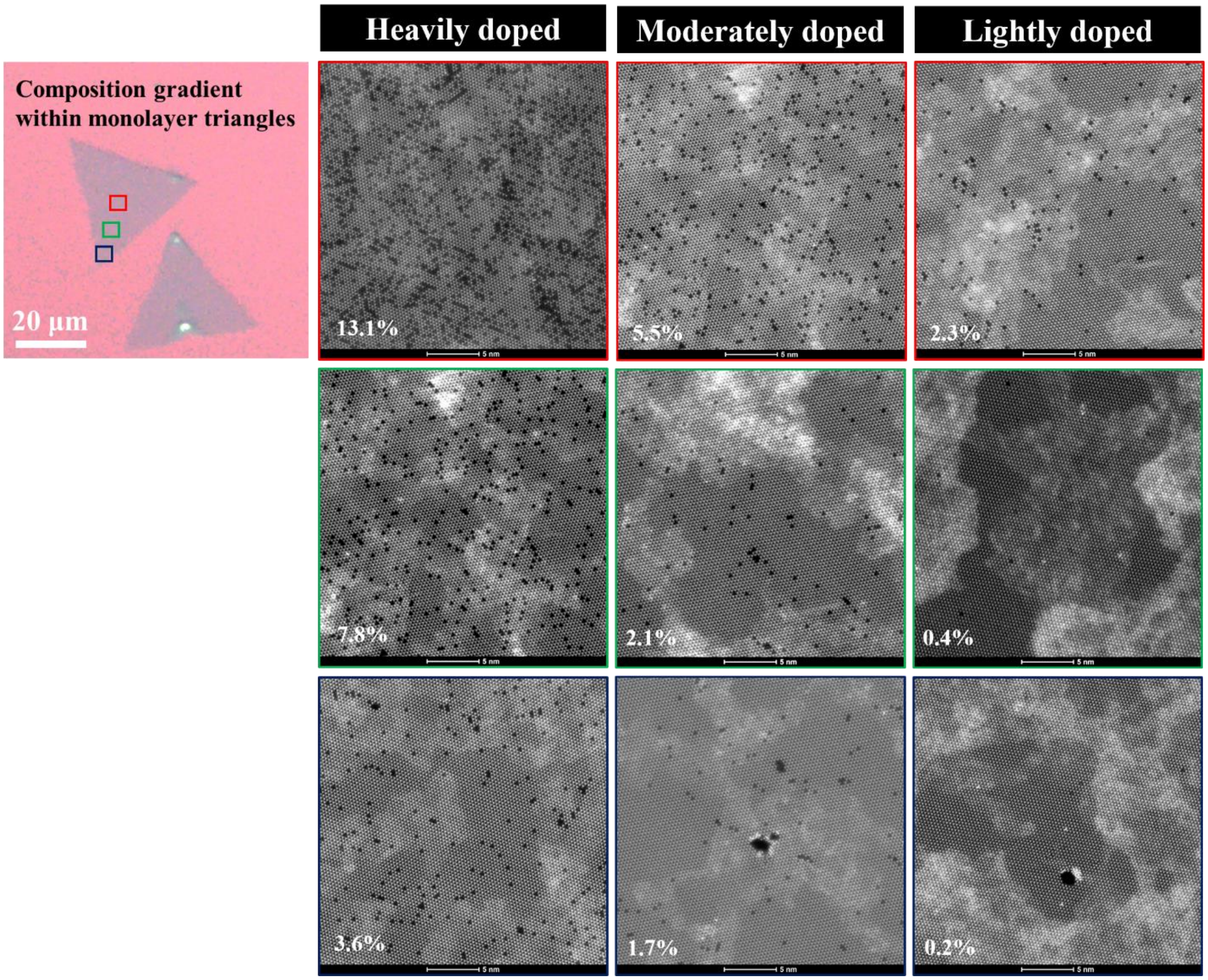

Figure S4 Doping concentration gradients within V-doped monolayer WS_2 triangles, local vanadium doping concentrations extracted from HAADF-STEM images at regions of center (red), middle (green), and edge (blue) of the triangles, respectively.

4. Raman Spectroscopy of pristine and V-doped WS_2 monolayers.

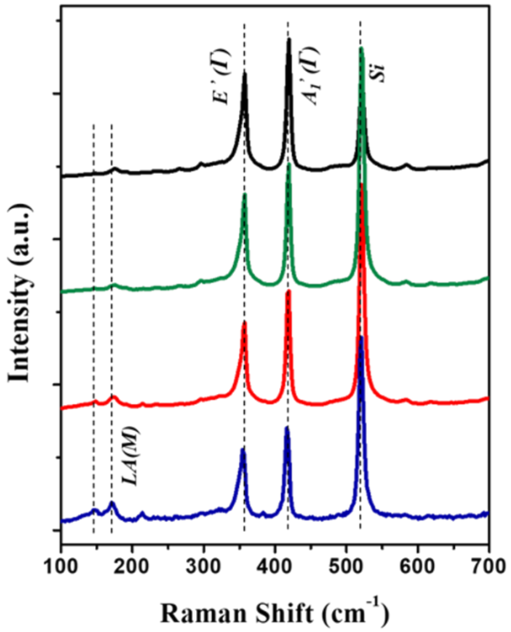

Peak position (cm^{-1}) @488 nm	E' (cm^{-1})	A_1' (cm^{-1})
pristine	357.6	419.3
Lightly doped	357.3	418.9
Moderate doped	356.2	417.7
Heavily doped	353.4	416.0
**Peak position (cm^{-1}) @532 nm**	**E'+2LA(M) (cm^{-1})**	**A_1' (cm^{-1})**
pristine	352.8	419.6
Lightly doped	352.1	418.9
Moderate doped	351.9	418.6
Heavily doped	356.2	417.5

Figure S5 Raman spectra of pristine and V-doped WS_2 monolayers at 488 nm excitation laser. Table of E'(Γ) and A_1'(Γ) peak positions in Raman spectra.

5. Time-domain thermoreflectance measurements of pristine and V-doped WS_2 monolayers.

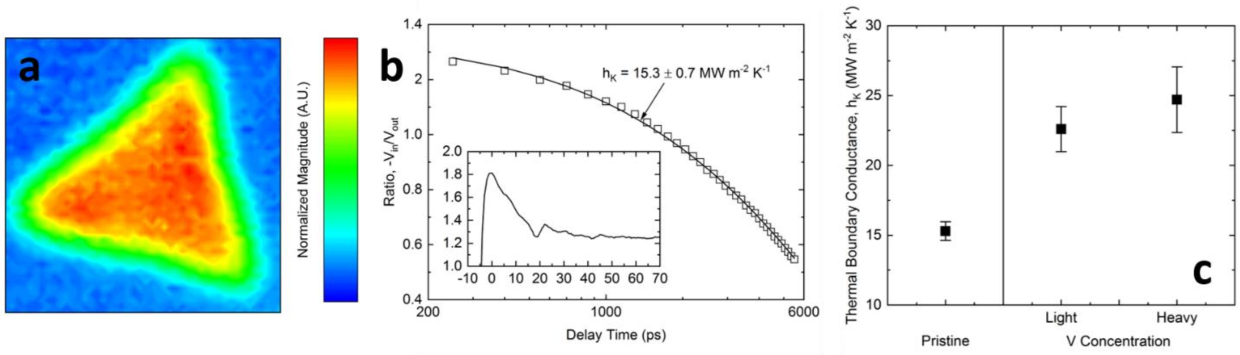

Figure S6 (a) Time-domain thermoreflectance (TDTR) magnitude micrograph of a pristine single-crystal WS_2 flake. (b) TDTR model and best fit for the conductance at the Al/pristine WS_2/SiO_2 interface. The inset shows the picosecond acoustics response at earlier time delays. (c) Results for the thermal boundary conductance at Al/doped WS_2/SiO_2 interfaces.

To examine the thermal boundary conductances (h_K) of devices contingent on the pristine and V-doped WS_2 monolayers, we measured the total conductance of the Al/doped WS_2/SiO_2 interface via time-domain thermoreflectance (TDTR) (detailed in Methods). An example of this magnitude micrograph can be seen in **Figure S6**(a) for a pristine WS_2 flake. The uniformity of the TR magnitude of the WS_2 flake suggests that the conductance is uniform. Full-time delay TDTR measurements near the center of the triangles were examined for pristine WS_2 and V-doped WS_2 samples. The TDTR curve and best fit are shown in Figure S6(b) for the pristine flake, where the inset shows the short delay time picosecond acoustic response, with which we used to extract the thickness of our Al transducer. This type of measurement was performed on lightly- (0.4%) and heavily-doped (8%) V-WS_2 crystals as well. The final results are shown in Figure S6(c) for all flakes measured, where an increase in thermal conductance of the Al/WS_2/SiO_2 interface was observed as the V doping concentration increased. The enhancement in conductance is correlated with the V substitutional sites in the WS_2 lattice. It is assumed that the inclusion of V dopants alters the local phonon density of states, allowing for an improved thermal conductance as the concentration of V is increased. Monolayer V-doped WS_2 with improved heat dissipation is promising as an electronic circuit component.

6. HAADF-STEM images and line profiles of V-doped WS_2 monolayers.

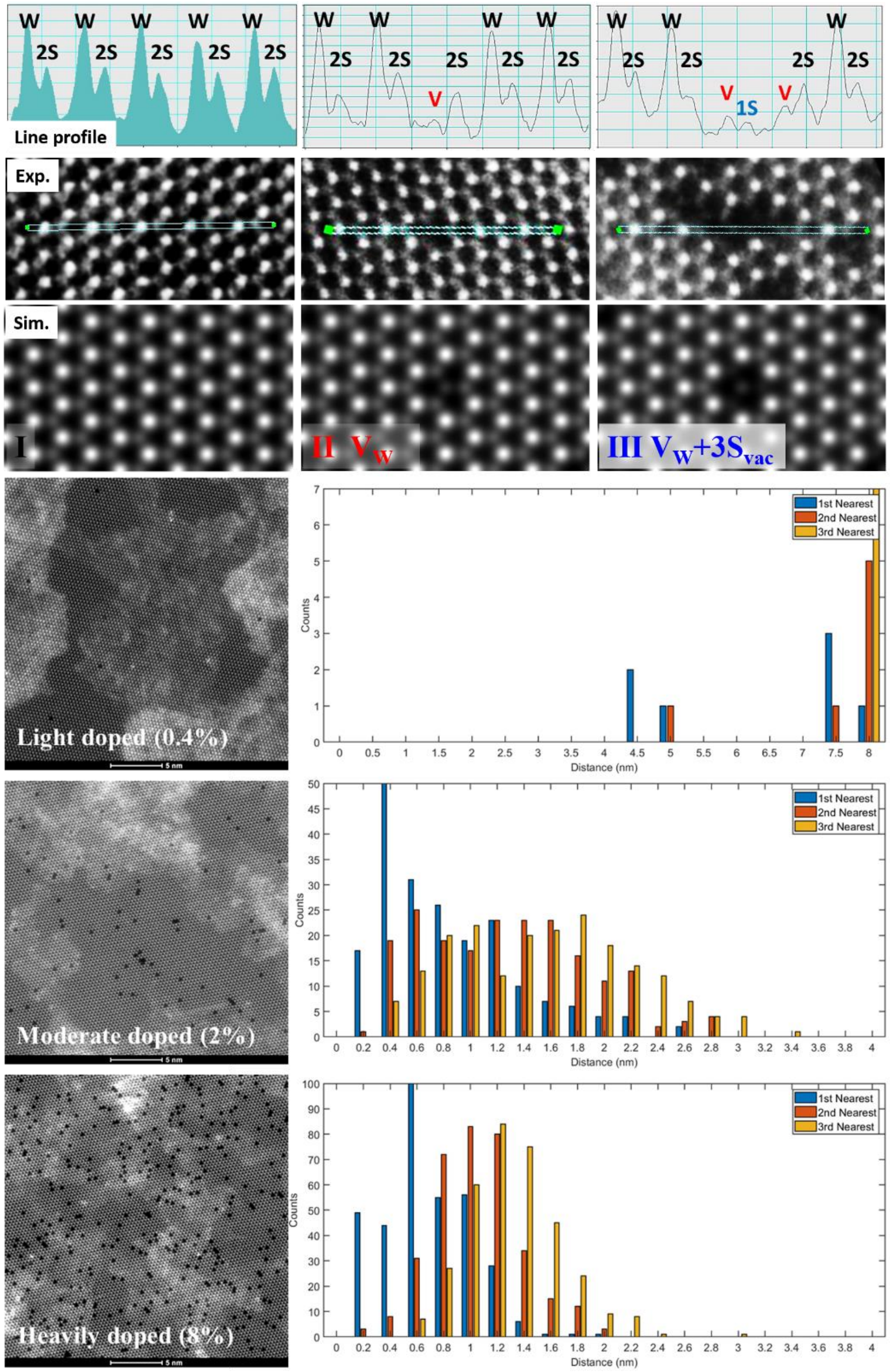

Figure S7 HAADF-STEM images and line profiles of V-doped WS_2. Upper: Intensity line profile of the experimental STEM image with pristine WS_2 lattice (black curve), V_W, and V_W+S_{vac}, respectively. Middle: corresponding experimental and simulated STEM images. Bottom: the corresponding histogram of the V-V near-neighbor distance distribution for V-doped WS_2 monolayers.

7. Background signals in VSM measurements.

It is always important to examine the background signal of the vibrating sample magnetometer (VSM) prior to any magnetic measurements, especially for samples having relatively weak magnetic signals. In this study, we measured the M-H dependence of both the SiO_2 substrate and the double side carbon tape used to hold up the pristine WS_2 and V-WSe_2 samples. As expected, we observe the diamagnetic signal for both the SiO_2 substrate and the tape (see **Figure S8**). This indicates that the ferromagnetic signals we recorded originate from the V-WS_2 samples.

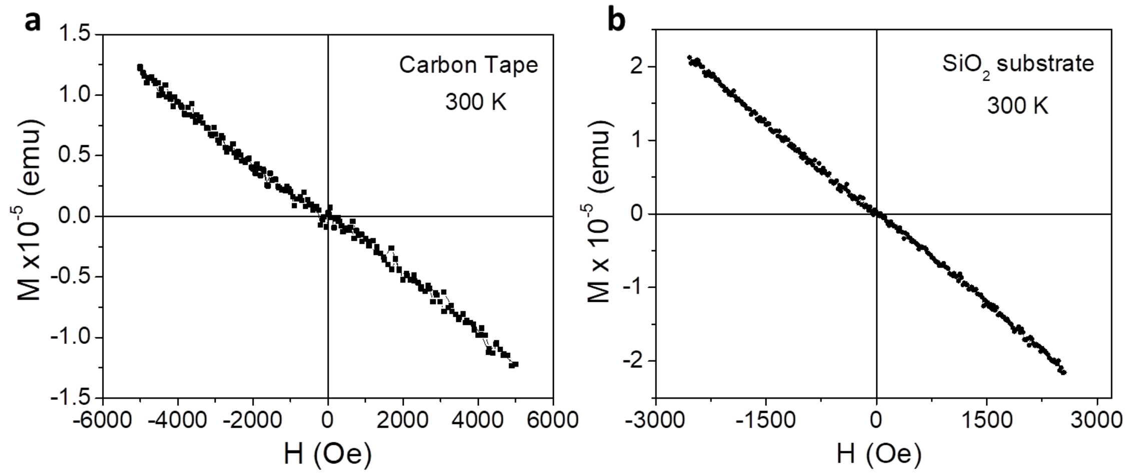

Figure S8 Magnetic field dependence of magnetization for (a) the carbon tape and (b) the SiO_2 substrate.

It has been shown that because subtracting diamagnetic backgrounds could cause unwanted effects on the M-H loops of 2D magnetic systems[4-6]. For instance, we show below the M-H loop of the V-doped WS_2 monolayer sample (**Figure S9**(a)) is preserved upon the data smoothing (Figure S9(b)) but is significantly modified after subtracting the diamagnetic background (Figure S9(c)). The smoothing method allows us to determine the values of M_S and H_C more precisely. This method has been accepted and used in the literature[4-6]. However, after subtracting the diamagnetic background, the M-H loop is deformed in shape, showing the unexpectedly smaller and larger values of H_C and M_S, respectively (see Figure S9(c)). To best reflect the magnetic characteristics of the V-WS_2 samples, in this manuscript, we show the as-measured M-H loops at 300K and deduce the saturation magnetization (M_S) and coercive field (H_C) directly from these loops after smoothing (the M-H loops are close to square when rotated to account for the diamagnetic background).

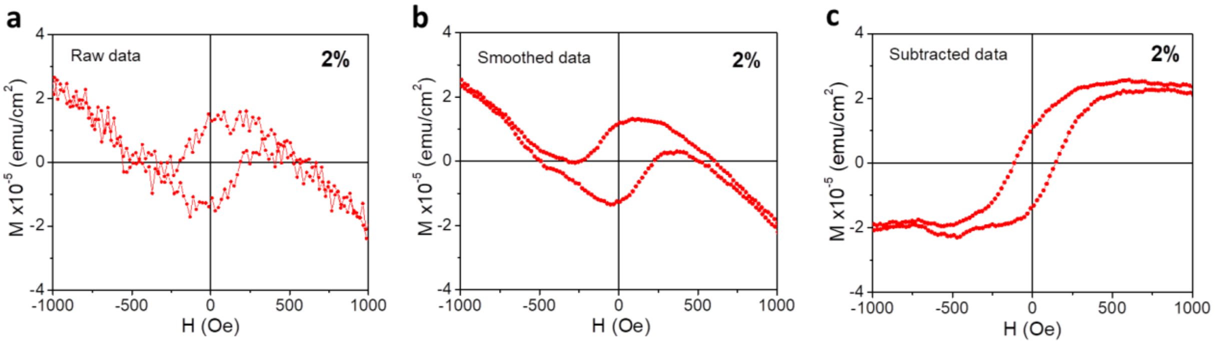

Figure S9 (a) As-measured, (b) smoothed, and (c) back-ground subtracted M-H loops of the 2at% V-doped WS_2 monolayer.

8. Reproducibility of magnetic measurements.

To check the reproducibility of the reported magnetic results, we performed magnetic measurements on three V-WS_2 samples for each concentration and obtained reproducible results. **Figure S10** shows the M-H loops taken at 300 K for the three 2at.% V-doped samples synthesized at different times. It is observed that the shape of the M-H loops is relatively identical. The M_S value is almost the same in all these cases. The slight difference in H_C could arise from S or W vacancies/defects that are differently present in these samples.

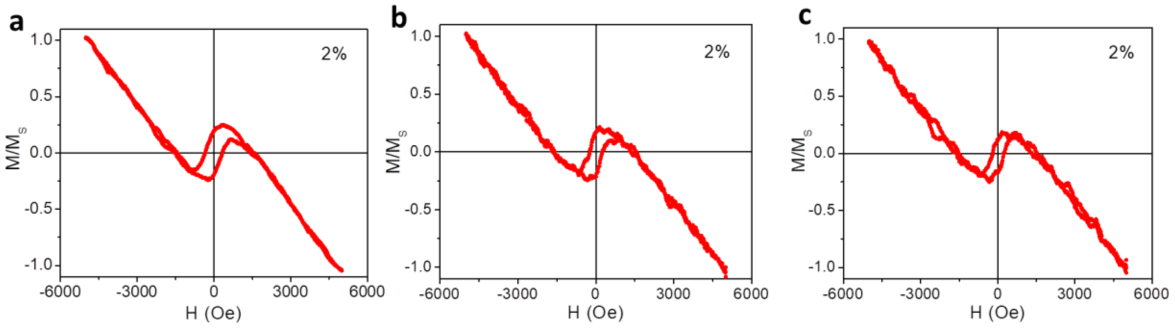

Figure S10 Three 2 at% V-doped samples synthesized at different times possess a similar M-H dependence, indicating the reproducibility of the reported magnetic results.

9. Temperature dependent magnetization data.

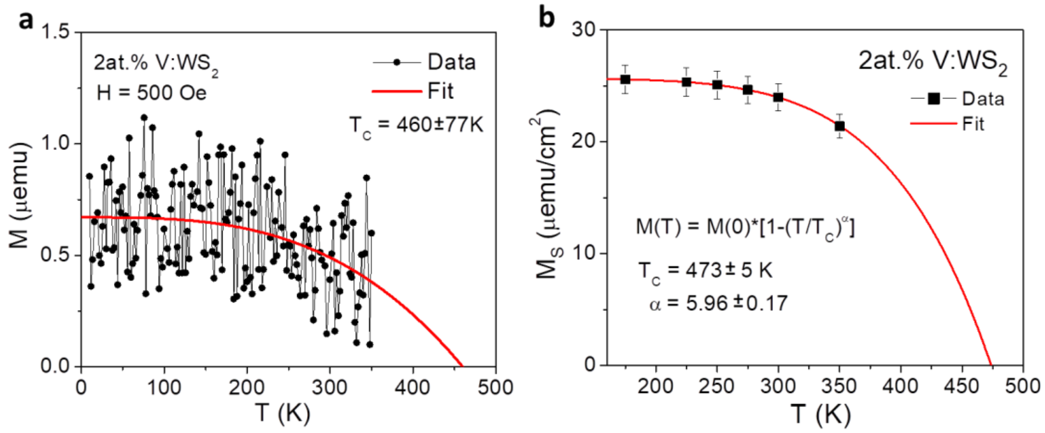

Figure S11 (a) Temperature dependence of magnetization (M) taken at a field of 500 Oe; (b) Temperature dependence of saturation magnetization (M_S) of the 2at% V-doped sample. The (red) solid line is a fit to the experimental data using $M(T) = M(0) \times [1-(T/T_C)^{\alpha}]$. The T_C values deduced from these fits are close. Some deviation could arise from the fact the M-T data were quite noisy.

10. Band structure of pristine and V-doped WS_2 monolayers.

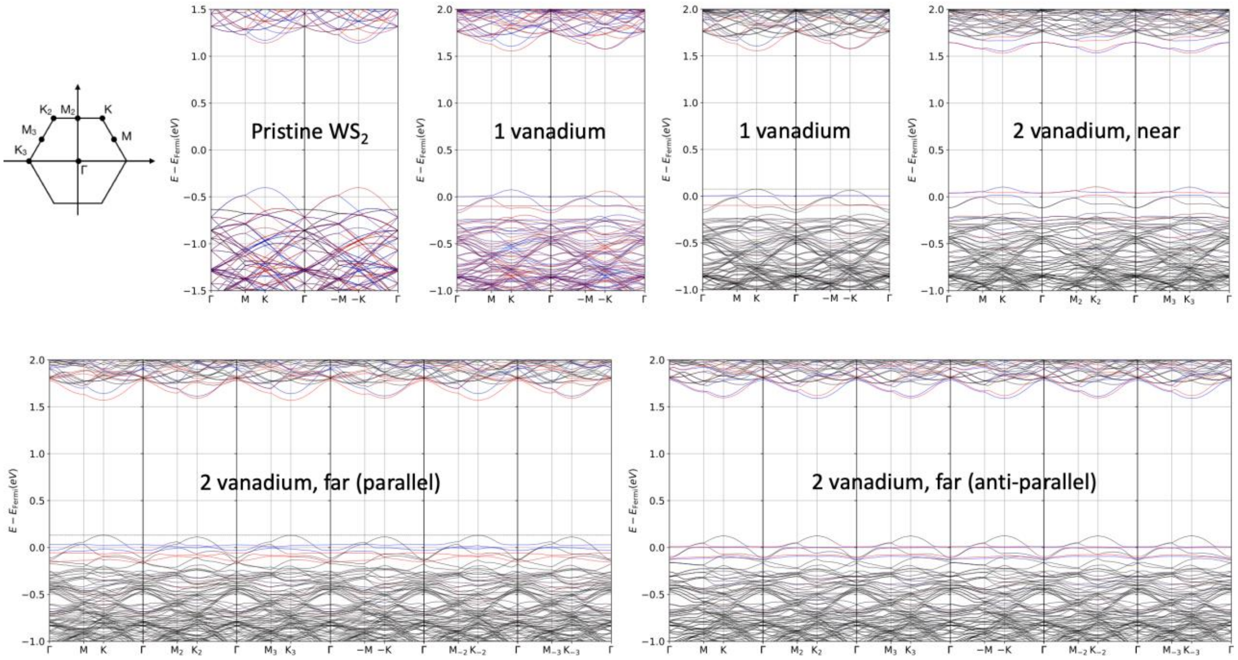

Figure S12 Band structures for 7×7 WS_2 supercell with different vanadium doping levels and dopant spin textures. The red/blue shows spin-up/down. For band structures with only several bands colored, the coloring is the projection onto vanadium d_{z^2} orbital to characterize the defect states. Along M-K-Γ, most bands flip the spin state while the defect states clearly remain the same spin state. The dashed line at the valance band top is to show the band maximum difference between K and –K.

In the band structure of pristine WS_2, similar to other work,[7] a giant 427 meV spin-orbit splitting at K (–0.401 eV and –0.828 eV) can be seen although it is folded. For the band structure of a single vanadium dopant, the spin splitting for the defect state is 0.10 eV, the valence band maximum difference between valleys is 12.6 meV, and the two spin states at the conduction band minimum in the –K valley are almost degenerate. For the band structure with 2 vanadium dopants at nearest-neighbor sites, the system converges to no spin polarization state, so both time-reversal and reflection symmetry (the "mirror" is between the two dopants) are preserved. For 2 vanadium dopants sitting farthest from each other with parallel spin directions, the valence band maximum differs by 19.5 meV between valleys, and the conduction band minima shift enough to have the same spin state among all valleys.

11. Estimation of the optimal doping level to obtain largest saturation magnetization

Suppose the doping level is p, i.e., the likelihood of a given metal site being V is p and $(1-p)$ for W. Starting from a given V dopant site, we assign half of the net magnetic moment given in Table S1 according to nearest dopant neighbor, assuming a random alloy. Specifically, the possibility of a V having its nearest dopant neighbor sitting at the 3rd nearest metal site is $(1-p)^{12}[1-(1-p)^{6}]$, and we assign 0.46 μ_B to it. The possibility of its nearest dopant neighbor sitting at a larger distance is $(1-p)^{18}$ and we assign a rough averaged moment in this region, 0.64 μ_B to it. Therefore, the expectation value of the moment contributed by a single V is roughly $0.46(1-p)^{12}[1-(1-p)^{6}]+0.64(1-p)^{18}$. The effective moment doping level will then be $p\{0.46(1-p)^{12}[1-(1-p)^{6}]+0.64(1-p)^{18}\}$, whose maximum occurs at $p \approx 7\%$ with a corresponding magnetization of 2×10^{-3} μ_B/Å^2. This simple estimate assumes an ideal random-alloy distribution of vanadium and a magnetic moment contribution based purely on dopant interactions with its nearest neighbor. However it gives a rough upper limit for optimal doping level, since the interaction with more neighbors will likely make the defect states more dispersive and reduce the net moments.

As an illustrative comparison, we present below the magnetic properties calculated without spin-orbit coupling.

Table S1 Energy and net magnetic moment for two vanadium atoms in a supercell (no spin-orbit coupling)

The energies are relative to that of the pair at the nearest distance. (★) shows the result is not spin-polarized. The antiferromagnetic state has a lower energy when neglecting spin-orbit coupling, and the spin-split dopant levels separate from the valence band sufficiently to attain integral occupancy. The general effect of quenching of the moment at close dopant separations is preserved even in the absence of spin-orbit interaction.

Dopant pair in lattice coords (Å)	Pair separation (Å)	Energy of the most stable spin texture (meV)	Net magnetic moment (μ_B)	Energy of competing spin texture (meV)	Moment of competing spin texture (μ_B)
**–1 0**	3.19	0 (★)	0.00	--	--
**1 1**	5.52	86.1 (★)	0.00	--	--
**0 2**	6.38	94.1 (★)	0.00	--	--
**1 2**	8.44	123.4 (⇅)	0.00	144.6 (⇈)	2.00
**0 3**	9.57	132.1 (⇅)	0.00	142.5 (⇈)	2.00
**2 2**	11.02	133.1 (⇅)	0.00	137.4 (⇈)	2.00
**1 3**	11.49	132.6 (⇅)	0.00	137.1 (⇈)	2.00

12. The spin polarization effect on band of V-doped WS_2 monolayers.

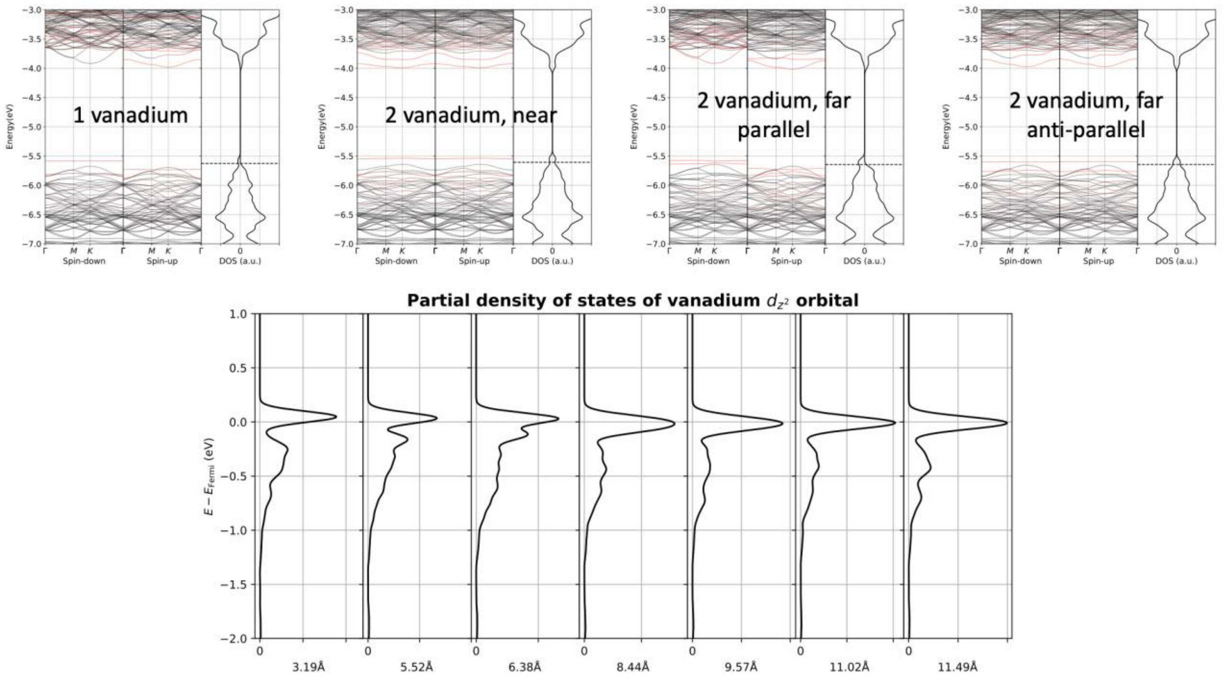

Figure S13 Band structures without spin-orbit coupling and the partial density of states without spin polarization. The red color of the bands represents a projection onto vanadium d_{z^2} orbital to characterize the defect states. The vacuum level has been set to 0. The spin-polarization is turned off for the partial density of states to get the bonding/anti-bonding splitting.

Without spin-orbit coupling, the vanadium is a shallow dopant. From the one vanadium case, the spin splitting (0.138 eV) of the defect state brings one spin state down to the valence band to become fully occupied while the other spin state is fully unoccupied, resulting in an integral Bohr magneton polarization. For the case of two vanadium atoms sitting at nearest-neighbor sites, the system shows no spin polarization. Anti-bonding state is fully unoccupied, and the bonding state hybridizes with WS_2 valence bands. For 2 vanadium dopants sitting farthest from each other with parallel spin directions, the two spin-down defect states are fully unoccupied, resulting in 2 Bohr magnetons of net magnetization. If they have anti-parallel spins, the two spin branches are nearly degenerate, and the up and down spin states center at different dopant sites.

The quenching of the magnetic moments in the cases without spin-orbit coupling could be understood by comparing the bonding/anti-bonding splitting and the spin splitting of the defect state. If the bonding/anti-bonding splitting is larger, the moments are quenched. We choose d_{z^2} to

track the defect state, since the single vanadium defect state projects only onto the V d_{z^2} orbital (and Mo and S orbitals). The bonding/anti-bonding energy splitting is 0.31, 0.20, and 0.15 eV for the closest three separations, beyond which the splitting is not resolved (<0.1 eV) due to the 0.05 eV Gaussian smearing. For comparison, the spin splitting is 0.138 eV. The pair of bonding/antibonding levels is most apparent at 5.52 and 6.38 Å separations (for 3.19 Å, the lower defect state sufficiently splits that it overlaps with other valence band states).

13. Dependence of magnetic coupling on other factors

Table S2 Energy difference (meV) between parallel and anti-parallel spin texture for 2 vanadium dopants with space separation 8.44 Å under different calculation settings.

1 hole	0.5 hole	Intrinsic	0.5 electron	1 electron	U = 1 eV	2 sulfur vacancies
13.9	–3.6	–13.3	–0.2	--	–4.7	–1.5

Negative digits mean that parallel spin texture is preferred and vice versa. The U parameter used for DFT+U method is taken from reference [8]. The two sulfur vacancies are sitting next to each vanadium dopants. For 1 electron doping case, only the parallel state can be stabilized, and the anti-parallel state is not a local minimum.

References

[1] F. Zhang, Y. Lu, D. S. Schulman, T. Zhang, K. Fujisawa, Z. Lin, Y. Lei, A. L. Elias, S. Das, S. B. Sinnott, *Science advances* **2019**, *5*, eaav5003.

[2] X. Zhang, Z. Y. Al Balushi, F. Zhang, T. H. Choudhury, S. M. Eichfeld, N. Alem, T. N. Jackson, J. A. Robinson, J. M. Redwing, *Journal of Electronic Materials* **2016**, *45*, 6273.

[3] S. J. Yun, D. L. Duong, D. M. Ha, K. Singh, T. L. Phan, W. Choi, Y.-M. Kim, Y. H. Lee, *Advanced Science* **2020**, *7*, 1903076.

[4] P. M. Coelho, H. P. Komsa, K. Lasek, V. Kalappattil, J. Karthikeyan, M. H. Phan, A. V. Krasheninnikov, M. Batzill, *Advanced Electronic Materials* **2019**, *5*, 1900044.

[5] D. J. O'Hara, T. Zhu, A. H. Trout, A. S. Ahmed, Y. K. Luo, C. H. Lee, M. R. Brenner, S. Rajan, J. A. Gupta, D. W. McComb, *Nano Lett.* **2018**, *18*, 3125.

[6] D. J. O'Hara, T. Zhu, R. K. Kawakami, *IEEE Magnetics Letters* **2018**, *9*, 1.

[7] Z. Zhu, Y. Cheng, U. Schwingenschlögl, *Physical Review B* **2011**, *84*, 153402.

[8] H. L. Zhuang, R. G. Hennig, *Phys. Rev. B* **2016**, *93*, 054429.
